# Space scan statistics to identify clusters of neonatal mortality associated with bacterial sepsis

**DOI:** 10.1017/S0950268824001663

**Published:** 2024-12-13

**Authors:** Daniela Testoni Costa-Nobre, Ana Sílvia Scavacini Marinonio, Milton Harumi Miyoshi, Adriana Sanudo, Kelsy Catherina Nemo Areco, Mandira Daripa Kawakami, Rita de Cássia Xavier Balda, Tulio Konstantyner, Carina Nunes Vieira e Oliveira, Paulo Bandiera-Paiva, Rosa Maria Vieira de Freitas, Monica La Porte Teixeira, Bernadette Waldvogel, Maria Fernanda de Almeida, Ruth Guinsburg, Carlos Roberto Veiga Kiffer

**Affiliations:** 1 Escola Paulista de Medicina, Universidade Federal de São Paulo, São Paulo, Brazil; 2 Gerência Demográfica da Fundação Sistema Estadual de Análise de Dados (Fundação SEADE), São Paulo, Brazil

**Keywords:** clusters, disease, neonatal mortality, neonatal sepsis, spatial analysis

## Abstract

Our study aim was to identify high-risk areas of neonatal mortality associated with bacterial sepsis in the state of São Paulo, Southeast Brazil. We used a population-based study applying retrospective spatial scan statistics with data extracted from birth certificates linked to death certificates. All live births from mothers residing in São Paulo State from 2004 to 2020 were included. Spatial analysis using the Poisson model was adopted to scan high-rate clusters of neonatal mortality associated with bacterial sepsis (WHO-ICD10 A32.7, A40, A41, P36, P37.2 in any line of the death certificate). We found a prevalence of neonatal death associated with bacterial sepsis of 2.3/1000 live births. Clusters of high neonatal mortality associated with bacterial sepsis were identified mainly in the southeast region of the state, with four of them appearing as cluster areas for all birth weight categories (<1500 g, 1500 to <2500 g and ≥ 2500 g). The spatial analysis according to the birth weight showed some overlapping in the detected clusters, suggesting shared risk factors that need to be explored. Our study highlights the ongoing challenge of neonatal sepsis in the most developed state of a middle-income country and the importance of employing statistical techniques, including spatial methods, for enhancing surveillance and intervention strategies.

Neonatal sepsis is a major health problem, affecting newborns worldwide and causing a significant number of deaths. It is estimated that every year, around 3 million newborns develop sepsis, with death rates ranging from 11% to 19%. In a systematic review examining the incidence of sepsis in neonates and children, data were collected from 22601 newborns across high- and middle-income countries. The findings indicated that the incidence of neonatal sepsis in middle-income countries can be up to 40 times higher, with mortality rates up to twice as high compared to those in high-income countries [1]. Nevertheless, sepsis remains one of the leading causes of preventable childhood mortality in both settings. Data of low- and middle-income countries (LMIC) are still scarce and greatly unreliable, posing a challenge to estimate the disease prevalence and burden in these regions [1].

In recent years, the utilization of secondary data and advanced statistical techniques has surfaced as a promising approach for enhancing the epidemiological knowledge on neonatal sepsis surveillance. The utilization of spatial methods in this context holds promise in finding areas of heightened risk for this critical condition [2]. By leveraging spatial and data analytics, this approach stands poised to highlight patterns and clusters of neonatal sepsis incidence across geographical regions. These methods not only aid in defining high-risk areas but also help to elucidate the potential underlying causes driving the occurrence of neonatal sepsis. Therefore, our aim was to identify high-risk areas of neonatal mortality associated with bacterial sepsis in the state of São Paulo, Brazil, by using a spatial scan method, helping to unveil underlying determinants of this critical condition.

We used a population-based study applying retrospective spatial scan statistics to identify high-risk areas of neonatal mortality associated with bacterial sepsis occurrence in the State of São Paulo, Southeast Brazil. The database was built with information extracted from birth certificates linked to death certificates using deterministic linkage. All live births from mothers residing in São Paulo State from 2004 to 2020 were included. Live births less than 22 weeks’ gestation and those with unknown birth weight were excluded. Neonatal deaths associated with bacterial sepsis were defined as any death between 0 and 27 days after birth with World Health Organization International Disease Codes 10^th^ed. (WHO-ICD10) A32.7, A40.0-A40.9, A41.0 – 41.5, P36.0-P36.9, P37.2 in any line of the death certificate. The State of São Paulo has an area of 248220 km^2^, with a population of approximately 44 million people within 645 municipalities and the highest Human Development Index in Brazil (score of 0.783).

A spatial analysis using the discrete Poisson model was adopted to scan clusters with high rates of neonatal mortality associated with bacterial sepsis, using the aggregated mortality rate per municipality [3]. The rates for each scanning window were calculated based on the number of deaths and births in the municipalities whose centroids fell within the window. In order to assess the significance at a level of *p* < 0.01, Monte Carlo simulations with 999 replications were performed. To calculate the log-likelihood radio (LLR) and the corresponding p-value and relative risk (RR) for different scanning windows, the Poisson probability model with a circular scanning window with a maximum cluster size of 30% of the population at risk and 50 km was used [4]. The minimum number of cases per cluster was defined as 10.

From 2004 to 2020, there were 10265105 live births from mothers living in São Paulo State. We excluded 3491 with gestational age less than 22 weeks and 410000 with unknown birth weight. Were included in the study 9851614 live births (138879 (1.4%) <1500 g birth weight, 766581 (7.8%) 1500 g to <2500 g birth weight and 8946144 (90.8%) ≥2500 g birth weight). A total of 23606 infants died with WHO-ICD-10 codes of bacterial sepsis in the death certificate (2.3 per 1000 live births). Among these deaths, 21% (*n* = 4955) occurred within 72 h, 25% (5980) between 72 h and 6 days after birth, and 54% (12751) between 7 and 27 days after birth.

The main maternal characteristics of neonates who died with bacterial sepsis were: age 20–35 years in 64%, schooling ≥8 years in 71%, and ≥ 4 prenatal visits in 76%. Among the neonates, 56% were male, the birth weight < 1500 g in 64%, 1500–2499 g in 19%, and ≥ 2500 g in 17%. The Apgar scores in the 5th minute were < 2 in 5%, 2–6 in 33%, and 7 or more in 62%.

Neonatal bacterial sepsis-associated deaths were not randomly distributed over the state, and clusters of high neonatal mortality associated with bacterial sepsis were identified mainly in the southeast region of the state (Figure 1). The regions of Sorocaba, Baixada Santista, Campinas, Taubaté, and São Paulo Metropolitan Area appeared as cluster areas for all birth weight categories.

**Figure 1.**
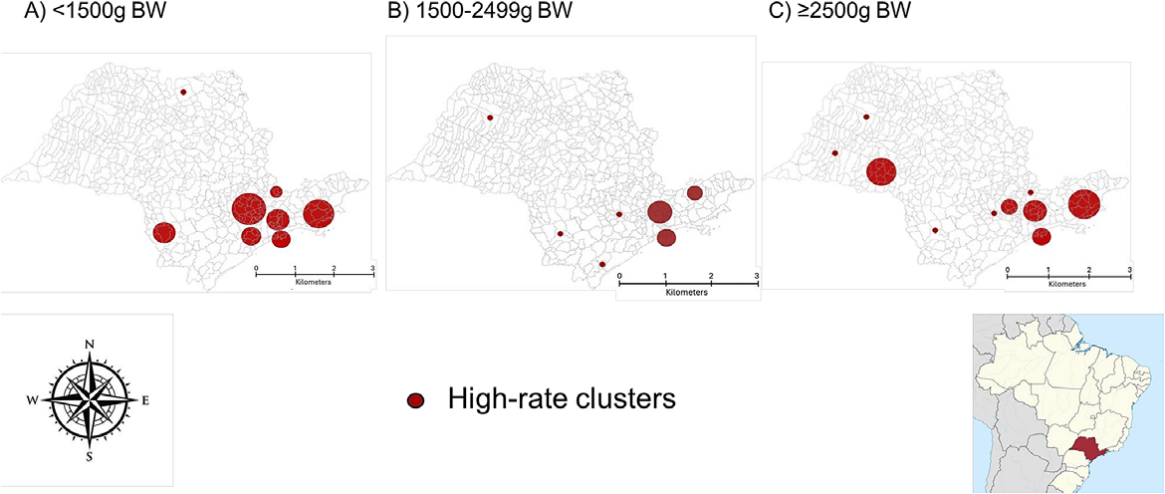
Bacterial sepsis neonatal deaths clusters, according to birth weight (BW). Clusters in the following municipalities: (a) Sorocaba, Baixada Santista, Campinas, Taubaté, Grande São Paulo. (b) Sorocaba, Baixada Santista, Campinas, Taubaté, Grande São Paulo, Araçatuba, Bauru, Presidente Prudente, Registro. (c) Sorocaba, Baixada Santista, Campinas, Taubaté, Grande São Paulo, Marília, Presidente Prudente, São José do Rio Preto.

We found a rate of neonatal mortality associated with bacterial sepsis of 2.3 per 1000 live births. The incidence of the disease varies across different income settings. While high-income countries report incidence rates ranging from 1 to 12 per thousand live births, LMICs face a higher burden of the disease. It is estimated that 15–30% of infants with neonatal sepsis will die before discharge [5, 6].

Conducting epidemiological research in middle-income countries often involves the analysis of secondary data sources, such as health records, surveillance systems, and national databases. While challenges related to data quality and completeness exist, numerous studies have successfully utilized secondary data to investigate infectious diseases [7]. Recent national studies estimate a burden of 456921 hospitalizations and 245483 deaths associated with sepsis in São Paulo State between 2013 and 2020 among general hospitalizations [8, 9]. Our study focused solely on neonatal sepsis and covered a slightly different period (2004–2020). Notably, in our results, the estimated burden included 23606 neonatal deaths associated with bacterial sepsis, underscoring the significant impact of the neonatal period on overall sepsis-associated lethality.

The methodology applied allowed us to identify hotspots of neonatal mortality associated with bacterial sepsis. Spatial approaches like scan statistics offer valuable tools for detecting clusters of disease cases within space and time, assisting in directing the allocation of resources and interventions [10]. We identified common areas of high neonatal mortality associated with bacterial sepsis for the three different birth weight ranges. Although this study does not focus on root cause analysis of the clusters, it is well known that regions identified with common clusters of bacterial sepsis-associated neonatal mortality across different birth weight ranges often contain highly specialized neonatal care units. This suggests potential flaws in the health regionalization system for specialized neonatal care, as sepsis cases appear to be more prevalent in these municipalities. However, the regionalization system’s deficiencies may not be the sole explanation for these clusters. The high mortality clusters might also result from broader health system infrastructure issues, such as patient-to-professional ratios, hospital resources, standard of care disparities in the studied regions, or lower social determinants of health (lower income level, education, sanitary conditions and access to resources, for example). On the other hand, the higher mortality areas may also reflect the concentration of severe cases due to referrals to most specialized hospitals. These hypotheses suggest directions for future studies on causal cluster analysis, which could inform and improve health policies.

There are some limitations to our methodology. Scan statistics detect clusters based on the geographic dependency of cases but may miss clusters that do not fit the predefined window. Additionally, the size and location of detected clusters may vary depending on input parameters, such as window size. Thus, subjectivity in parameter selection might induce bias or lead to misinterpretation. Another limitation is that spatial methods do not fully account for the explanation of the condition since other non-geographic factors may subside and may interfere in clusters. The results of the analysis often depend on the quality of the data, and underreporting of sepsis diagnoses on death certificates may affect the reliability of cluster detection.

In conclusion, the present study estimated with a quite restrictive method (IC10 codes with exclusive indication of bacterial sepsis) that 23606 infants died with bacterial sepsis diagnosis from 2004 to 2020 in the State of São Paulo, with a neonatal mortality rate of 2.3 per 1000 live births. These deaths were not randomly distributed over the state. The spatial analysis according to the birth weight of neonates who died with bacterial sepsis showed some overlapping in the detected clusters, suggesting shared risk factors that need to be explored. Our study highlights the ongoing challenge of neonatal sepsis in the most developed state of a middle-income country and the importance of employing statistical techniques, including spatial methods, for enhancing surveillance and intervention strategies. The identified high-risk clusters of neonatal deaths associated with bacterial sepsis in São Paulo State, Brazil, may provide valuable insights for future root-cause analysis studies in these regions and potentially help the planning of targeted resource allocation and interventions.

## Data Availability

The data that support the findings of this study will be available in Zenodo at https://zenodo.org/uploads/12696458 following a embargo. On 1 September, the files will automatically be made publicly accessible.
